# Molecularly targeted nanoparticles: an emerging tool for evaluation of expression of the receptor for advanced glycation end products in a murine model of peripheral artery disease

**DOI:** 10.1186/s11658-021-00253-0

**Published:** 2021-03-16

**Authors:** Marcin Woźniak, Christian J. Konopka, Agata Płoska, Jamila Hedhli, Anna Siekierzycka, Maciej Banach, Rafal Bartoszewski, Lawrence W. Dobrucki, Leszek Kalinowski, Iwona T. Dobrucki

**Affiliations:** 1 Department of Medical Laboratory Diagnostics – Fahrenheit Biobank BBMRI.pl, Medical University of Gdansk, 7 Debinki Street, 80–211, Gdansk, Poland; 2 University of Illinois at Urbana-Champaign Beckman Institute for Advanced Science and Technology, 405 N Mathews Ave, MC-251, 61801 Urbana, IL USA; 3 Department of Bioengineering, University of Illinois at Urbana-Champaign, Urbana, IL USA; 4 Biobanking and Biomolecular Resources Research Infrastructure Poland (BBMRI.pl), Gdansk, Poland; 5 Cancer Center at Illinois, University of Illinois at Urbana-Champaign, Urbana, IL USA; 6 Carle-Illinois College of Medicine, University of Illinois at Urbana-Champaign, Urbana, IL USA; 7 Department of Hypertension, Medical University of Lodz, Lodz, Poland; 8 Department of Biology and Pharmaceutical Botany, Medical University of Gdansk, Gdansk, Poland; 9 BioTechMed Centre, Department of Mechanics of Materials and Structures, Gdansk University of Technology, Gdansk, Poland

**Keywords:** RAGE, AGEs, Molecular imaging, Ischemia

## Abstract

**Background:**

Molecular imaging with molecularly targeted probes is a powerful tool for studying the spatio-temporal interactions between complex biological processes. The pivotal role of the receptor for advanced glycation end products (RAGE), and its involvement in numerous pathological processes, aroused the demand for RAGE-targeted imaging in various diseases. In the present study, we evaluated the use of a diagnostic imaging agent for RAGE quantification in an animal model of peripheral artery disease, a multimodal dual-labeled probe targeted at RAGE (MMIA-CML).

**Methods:**

PAMAM dendrimer was conjugated with Nε-carboxymethyl-lysine (CML) modified albumin to synthesize the RAGE-targeted probe. A control untargeted agent carried native non-modified human albumin (HSA). Bifunctional *p*-SCN-Bn-NOTA was used to conjugate the ^64^Cu radioisotope. Surgical right femoral artery ligation was performed on C57BL/6 male mice. One week after femoral artery ligation, mice were injected with MMIA-CML or MMIA-HSA labeled with ^64^Cu radioisotope and 60 min later in vivo microPET-CT imaging was performed. Immediately after PET imaging studies, the murine hindlimb muscle tissues were excised and prepared for gene and protein expression analysis. RAGE gene and protein expression was assessed using real-time qPCR and Western blot technique respectively. To visualize RAGE expression in excised tissues, microscopic fluorescence imaging was performed using RAGE-specific antibodies and RAGE-targeted and -control MMIA.

**Results:**

Animals subjected to PET imaging exhibited greater MMIA-CML uptake in ischemic hindlimbs than non-ischemic hindlimbs. We observed a high correlation between fluorescent signal detection and radioactivity measurement. Significant RAGE gene and protein overexpression were observed in ischemic hindlimbs compared to non-ischemic hindlimbs at one week after surgical ligation. Fluorescence microscopic staining revealed significantly increased uptake of RAGE-targeted nanoparticles in both ischemic and non-ischemic muscle tissues compared to the control probe but at a higher level in ischemic hindlimbs. Ischemic tissue exhibited explicit RAGE dyeing following anti-RAGE antibody and high colocalization with the MMIA-CML targeted at RAGE.

**Conclusions:**

The present results indicate increased expression of RAGE in the ischemic hindlimb and enable the use of multimodal nanoparticles in both in vitro and in vivo experimental models, creating the possibility for imaging structural and functional changes with a RAGE-targeted tracer.

**Supplementary Information:**

The online version contains supplementary material available at 10.1186/s11658-021-00253-0.

## Introduction

The dynamic development of biomedical techniques has led to the formation of a new field of science called molecular imaging. Molecular imaging could be explained as a non-invasive insight of a natural phenomenon in real time at the molecular and cellular level in the body, tissues, or within cell cultures. In general, molecular imaging requires the use of specialized equipment alone (label-free imaging) or in combination with molecular probes that allow imaging of the biochemical phenomena within specific tissues and organs. Obtained data can help better understand human and animal biology, identify existing pathologies, and provide information on disease pathomechanisms. Molecular imaging has a great potential in developing novel targeted diagnostics, monitored therapy, discovering new drugs, and understanding various biological processes at the nanoscale, such as protein-protein interactions and enzymatic transformations [1–4].

### Microscopic imaging techniques

Imaging cells and tissues with light is considered a relatively effortless research method widely used in basic sciences and routine diagnostic processes. Since the invention of the first microscope in 1674 by Anton van Leeuwenhoek, several useful microscopic techniques have been developed, such as light and darkfield microscopy combined with interference-difference contrast (improves resolution, provides a three-dimensional optical effect). In parallel with fluorescence microscopy development, many macroscopic optical imaging techniques have emerged. Macroscopic optical imaging methods allow scientists to carry out non-invasive imaging of small animals’ entire body using a large field of view (from a few millimeters to several centimeters) [5]. Fluorescent and bioluminescent techniques are examples of optical macroscopic imaging techniques.

### Macroscopic imaging techniques: optical and radioisotope based methods

Fluorescent and bioluminescent imaging equipment is quite affordable and relatively easy to install. These methods are considered relatively safe because the optical photon radiation energy is low compared to the gamma radiation emitted in positron emission tomography (PET) and single-photon emission computed tomography (SPECT). The use of low-energy radiation in combination with the lower optical wavelength of emitted light limits the penetration depth to a few millimeters. For this reason, it is practically impossible to analyze organs and tissues in humans and large animals without an endoscope allowing a maximum approach to the examined structures. However, many fluorescent and bioluminescent imaging probes (i.e., emitting higher wavelength light) can potentially be used to visualize the internal organs of small animals. Their small size makes optical imaging techniques suitable for preclinical studies [6]. However, it is important to recognize the limitations of optical imaging for quantitative analysis [7]. Molecular imaging of living organisms has strong connections with nuclear medicine. In the last decade, the analysis of biological processes using PET and SPECT techniques with new molecular probes specific to biological structures has become more common. Currently, it is not limited to the injection of radionuclides. One of the main goals of molecular imaging is to translate the strategy used in in vitro research into in vivo analysis to overcome existing technical limitations. It is extremely important to select the right imaging technique and synthesize an imaging probe containing a directional and signaling component. One of the potential applications of molecular imaging presented here is the quantitative imaging of the receptor for advanced glycation end products (RAGE). RAGE, which is expressed at a low level in homeostasis, is significantly overexpressed in numerous pathological conditions, e.g., in diseases of civilization such as diabetes, atherosclerosis, and cancer [8].

### RAGE receptor

The receptor for advanced glycation end products is a transmembrane multiligand receptor which belongs to the immunoglobulin superfamily, classified as a class J scavenger receptor [8–10]. RAGE is located on the surface of various cells, such as smooth muscle cells, hepatocytes, neurons, endothelial cells, and monocytes. Its expression may be decreased or induced in a number of human pathologies [11–14]. As the name implies, RAGE was first identified in bovine endothelial cells as a receptor binding advanced glycation end products (AGEs) [10]. Studies show the accelerated formation and accumulation of AGEs, particularly Nε-carboxymethyl-lysine (CML) in obesity – despite the absence of diabetes [15, 16]. Research from 2014 indicated a strong relationship between AGEs, RAGE, diabetes, and neurodegenerative diseases such as Alzheimer’s disease [17]. Currently, CML is the most examined AGE in RAGE activation and intracellular signal transduction [18]. Ligand binding to RAGE induces a signal transduction process responsible for the differential effect of RAGE on the expression of many genes [19–22]. It has been proven that RAGE is expressed on many types of cells and tissues, controlling many cellular processes, such as inflammation and immune response mechanisms, cell migration and proliferation, apoptosis, and autophagy. Hence, there is a need to develop an image-based strategy to non-invasively and quantitatively evaluate RAGE expression in biological systems.

To address this unfulfilled need and the clinical challenges, we have developed a technique to image RAGE expression non-invasively. We recently reported the use of a multimodal molecular imaging agent targeted to the RAGE receptor [23, 24].

This study aimed to demonstrate that our previously synthesized and characterized RAGE probe is suitable for both in vivo quantitative nuclear imaging and both in vitro and ex vivo analysis.

## Materials and methods

### Synthesis of RAGE-targeted probe

Multimodal (PET-optical) imaging agent (MMIA) was synthesized using a previously published method [24]. Briefly, RAGE-targeted and non-targeted probes used generation 4 polyamidoamine (PAMAM) dendrimer as the probe’s backbone. Firstly, PAMAM’s surface primary amines were modulated by acetylating the surface amines with sulfosuccinimidyl acetate. To obtain the synthesized probe’s fluorescence and radioactive properties, the chelator *p*-SCN-Bn-NOTA (for chelation of copper-64, ^64^Cu) and NHS-rhodamine were each conjugated to PAMAM. Finally, the construct was conjugated with the RAGE ligand CML crosslinked to PAMAM’s amine groups. The control probe was synthesized by conjugating unmodified human serum albumin (HSA) to PAMAM. During the synthesis, all intermediate products formed were refined employing 10 K or 100 K MWCO Amicon Ultra-15 filters (Millipore, USA) and lyophilized to achieve a crystalline solid.

### Cell culture

Our in vitro experimental model was human umbilical vein endothelial cells (HUVEC) acquired from the American Type Culture Collection (ATCC). The cell culture was conducted in T25 flasks or cell culture plates (Costar, USA), maintaining standard conditions: constant temperature of 37 °C and CO_2_ concentration of 5 %. Cells were grown in antibiotic-supplemented minimal essential medium, supplied with 10 % fetal bovine serum (FBS) and endothelial cell growth supplement. Accutase (Sigma-Aldrich, USA) was used for cells’ detachment and passaging. Experiments were performed on confluent HUVEC cultures at 4–5 passages.

### Cellular binding

in vitro assessment of RAGE probe binding was done using a previously described protocol [24]. Briefly, to induce the overexpression of RAGE, HUVEC cells cultured on 96-well plates at a density of 5 × 10^5^ cells/well were incubated in a medium enriched with 5.5 mM or 14 mM glucose for 12 h. Studies assessing the degree of RAGE probe binding were performed at various intervals and nanoparticle concentrations with constant final volume (100 µL) and temperature (37 °C). Before the experiments, a regular growth medium was substituted with PBS-based staining buffer containing 10 % FBS with pH = 7.4. A given quantity of targeted and non-targeted probes stained with a known fluorophore or labeled with radioisotope was utilized in the subsequent studies. Fluorescence signal intensity was evaluated using the Hybrid Multimode Microplate Reader (BioTek, USA) with excitation and emission wavelengths set to 520 nm and 550 nm, respectively; the radioactivity was measured using a gamma well counter (Perkin Elmer, USA).

### Preclinical model of PAD

Animal studies were performed according to the American Physiological Society’s guiding principles and sanctioned by the Institutional Animal Care and Use Committee at the University of Illinois at Urbana-Champaign. The murine model of hindlimb ischemia (HLi) was used to assess RAGE-targeted in vivo imaging feasibility with MMIA. HLi is currently the most common animal experimental model of peripheral artery disease (PAD) and is induced by the surgical ligation of the proximal and distal femoral arteries, resulting in blood flow impairment and hindlimb ischemia. The already verified protocol was utilized for surgical HLi induction in 8-12-week-old male C57BL/6 mice (purchased from Jackson Laboratories, USA) [25]. 1–3 % isoflurane was employed to anesthetize the animals and perform surgery and imaging. The procedure started with scissors making a 0.5-1.0 cm incision in the thigh’s medial part. The incision ran from the knee towards the abdomen. The femoral artery was dissected from the femoral vein and the femoral nerve using a surgical microscope. The femoral artery and its visible branches were ligated closer to the superficial epigastric artery and above the saphenous and popliteal arteries’ branching using silk sutures (Ethicon, USA). As a control, sham surgery was conducted on every mouse’s opposite leg. During placebo surgical intervention, the skin and muscle tissue were dismembered, but the artery persisted untouched. After the procedure, the animal was placed in a cage for recovery. A small postoperative wound and minimal inflammation were observed after surgery. All mice underwent RAGE-targeted micro positron emission tomography combined with computed tomography (microPET-CT) imaging at 1 week after surgical HLi.

### In vivo imaging

At one week after femoral artery ligation mice were injected intravenously through the jugular vein with approximately 4 MBq of either the targeted multimodal imaging agent conjugated with CML (MMIA-CML) (n = 6) or the control non-targeted multimodal imaging agent conjugated with HSA (MMIA-HSA) (n = 6) radiolabeled with ^64^Cu for microPET-CT imaging. One hour after radiolabeled probe injection, animals were subjected to the imaging session using a hybrid microPET-CT scanner (Inveon, Siemens Healthcare, USA) under the following conditions: static PET imaging continuing for 15 min with a 20 % energy window centered at 511 keV, accompanied by high-resolution anatomic computed tomography (CT). Following the imaging session, mice were directly sacrified, and hindlimbs muscle tissue fragments were collected for gene and protein analysis.

### RNA extraction, reverse transcription, and real‐time PCR

According to the manufacturer’s instructions, the RNeasy Fibrous Tissue Mini kit (Qiagen) was utilized to extract total RNA from murine muscle hindlimb tissue. An Epoch Microplate Spectrophotometer (BioTek, USA) was used to assess the integrity of isolated ribonucleic acid (RNA). In the subsequent steps, the extracted RNA was converted into deoxyribonucleic acid (DNA) in the reverse transcription process using a purchased kit (Sigma-Aldrich, USA), according to the manufacturer’s protocol. The RAGE messenger RNA expression pattern was assessed employing a real-time polymerase chain reaction (PCR) utilizing Sybr Green II Reaction Mix (Illumina Eco). Data were analyzed using the 2-^-^^ΔΔCt^ formula. The beta-actin protein coding gene (*ACTB*) and the glyceraldehyde 3-phosphate dehydrogenase protein coding gene (*GAPDH*) were used as reference genes. Amplification with simultaneous detection of the fluorescent signal was accomplished according to the following conditions: first primary denaturation and polymerase activation for 2 min at 95 °C, followed by 40 cycles of long denaturation for 5 s at 95 °C, then annealing for 20 s at 62 °C and elongation for 15 s at 72 °C.

The sequences of RAGE protein coding gene (*AGER*) primers were: 5ʹ-CACCCACCCTAGCCACGGACCTCAG-3ʹ and 5ʹ-CCAGCCCAGACTCACCCACAGAGCC-3ʹ.

Primers used for *ACTB* were: 5ʹ-GTCCACCCGCGAGCACAGCTTCTTT-3ʹ and 5ʹ-CTTTGCACATGCCGGAGCCGTTGTC-3ʹ.

Primers used for *GAPDH* were: 5ʹ-TCTTCCACCTTCGATGCCGGGGCTG-3ʹ and 5ʹ-TCCACCACCCTGTTGCTGTAGCCGT-3ʹ.

A particular reaction’s amplification efficiency was determined during the experiment planning using a standard curve created from six logarithmic serial dilutions. The efficiency measurement was reproducible and multiple standard curves were constructed. Target and reference genes were tested with serial dilutions. The outcomes were depicted with the logarithmic data for every dilution on the x-axis and Ct’s disparity (target-reference) for individual dilution on the y-axis. Individual amplicon melting curve analysis was performed to determine non-specific reaction product presence.

### Western blotting

Protein expression in muscle tissue sections was determined using Western blot. For protein extraction tissues were transferred to homogenization buffer (50 mM Tris-HCl, pH 7.8, 150 mM NaCl, 3 mM KCl, 2 mM ethylenediaminetetraacetic acid, 1 % sodium dodecyl sulfate, 1 % Triton, 1 mM dithiothreitol, 0.1 mM phenylmethylsulfonyl fluoride; 10 µg/mL protease inhibitor) and disintegrated in a laboratory homogenizer (T-10 basic ULTRA-TURRAX). Samples were then centrifuged at 12,000g for 15 min. The supernatant’s protein concentration was determined using the Bradford method in a Biotek Take3 microplate protein quantification. A Laemmli buffer with 5 % 2-mercaptoethanol buffer was added to 30 µg of protein, followed by boiling at 95 °C for 5 min. 30 µg of protein was loaded into each well. Sodium dodecyl sulfate–polyacrylamide gel electrophoretic separation was performed using a Mini-PROTEAN Tetra Cell electrophoresis apparatus (BioRad) on a 4–20 % gradient Mini-PROTEAN TGX gel (BioRad) at 80 V and 4 °C for 1.5 h or until bromophenol reached the end of the gel. Gels were then transferred to nitrocellulose membranes (BioRad) for 1.5 h at 150 mA and 4 °C. After the transfer, membranes were blocked for 16 h at 4 °C (3 % skimmed milk; 50 mM Tris-HCl, pH = 7.4; 150 mM NaCl; 0.1 % Tween 20). Following the blocking, membranes were kept with the primary anti-RAGE antibody diluted to a ratio of 1:1000 for 2 h at ambient temperature. After washing three times with tris buffered saline with Tween (TBST) buffer, membranes were kept in secondary antibody diluted in blocking buffer to a ratio of 1:2500 and conjugated with horseradish peroxidase for 1 h at ambient temperature and then rinsed three times with TBST again. Chemiluminescence identification was performed using a VisiGlo kit (Amresco, USA), and results were captured with a FusionFX (Vilber Lourmat). Signal quantification and protein normalization to ß-actin were performed using ImageJ software [26] (National Institutes of Health, USA).

### Immunofluorescence analysis

At 1 week after surgical ligation of the femoral artery, the mice tissue fragments were excised, samples were mounted in TissueTec (Sakura) and immediately frozen in methyl butane chilled to -150 °C. Then, ice-cold acetone fixed frozen sections (each 5 μm thick) were immersed into a buffer containing primary anti-RAGE antibody diluted to a ratio of 1:100 (Abcam, USA) and kept for 16 h at 4 °C. After washing, sections were incubated with secondary FITC-conjugated antibody (1:100, Abcam, USA) or/and MMIA-CML or MMIA-HSA for 2 h at ambient temperature, embedded with Fluoromount (Southern Biotech, USA), and imaged with an Olympus fluorescence microscope; images were processed with ImageJ software. According to algorithms formerly implemented by our group, the images were measured for the degree (percentage area) of positive staining in haphazardly selected high-driven (200×) regions.

### Statistical methods

We hypothesized that our previously synthesized and characterized probe is suitable for nuclear imaging and in vitro and ex vivo analysis. To verify our hypothesis, we first used the Shapiro–Wilk test to determine the data’s Gaussian distribution. Paired or unpaired two-tailed Student’s t-test was applied to establish significance among experimental groups. A *p*-value lower than 0.05 was deemed significant (*<0.05, **< 0.005). Results are depicted as mean ± mean squared error. To verify the relationship structure between a pair of quantitative variables, we performed a correlation test. We used a linear regression plot due to the Gaussian distribution of the data used and their linear distribution. To evaluate the strength of the relationship between the variables, we calculated Pearson’s coefficient.

## Results

The pivotal role of RAGE and its involvement in numerous pathological processes aroused the demand for RAGE-targeted imaging in various diseases. In the present study we evaluated the use of a diagnostic imaging agent for RAGE quantification in PAD, recently developed by our group, a multimodal dual-labeled probe targeted at RAGE (MMIA-CML).

### Assessment of probe’s multimodal properties

**Fig. 1:**
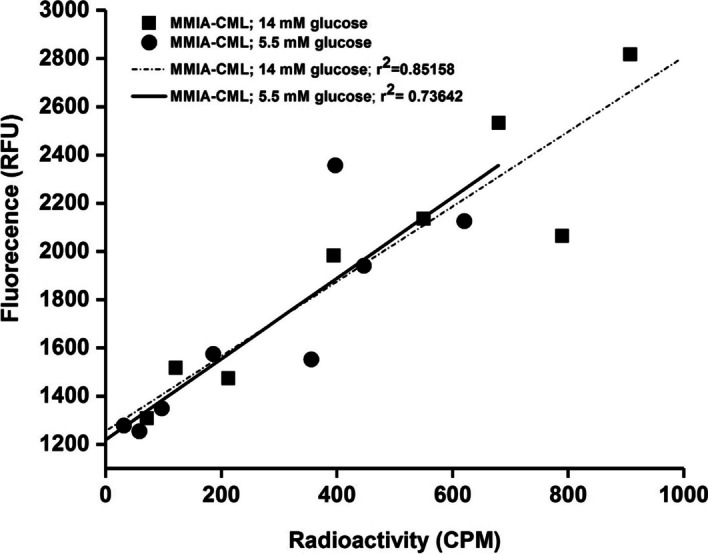
Correlation between fluorescence and gamma well-counting methods. Human umbilical vein endothelial cells (HUVEC) were cultured in a medium enriched with 5.5 mM, which mimics normoglycemic, or 14 mM glucose, which imitates hyperglycemic conditions in advance of the incubation with multimodal probes targeted at RAGE

In a previously published study, our synthesized targeted and non-targeted probes were extensively characterized in vitro [24]. In this study, we proceeded to test the correlation of RAGE targeting MMIA-CML using the nanoparticle’s multimodal properties in cell culture. HUVECs were incubated in normal or hyperglycemic conditions to induce RAGE overexpression. Conducted experiments demonstrated a high correlation between fluorescent signal detection and the radioactivity measurement. The correlation coefficient between two different experimental methods for cells cultured in medium containing 14 mM glucose was r^2^ = 0.85 while for HUVEC incubated with 5.5 mM glucose r^2^ = 0.74 (Fig. 1).

### In vivo RAGE expression

**Fig. 2:**
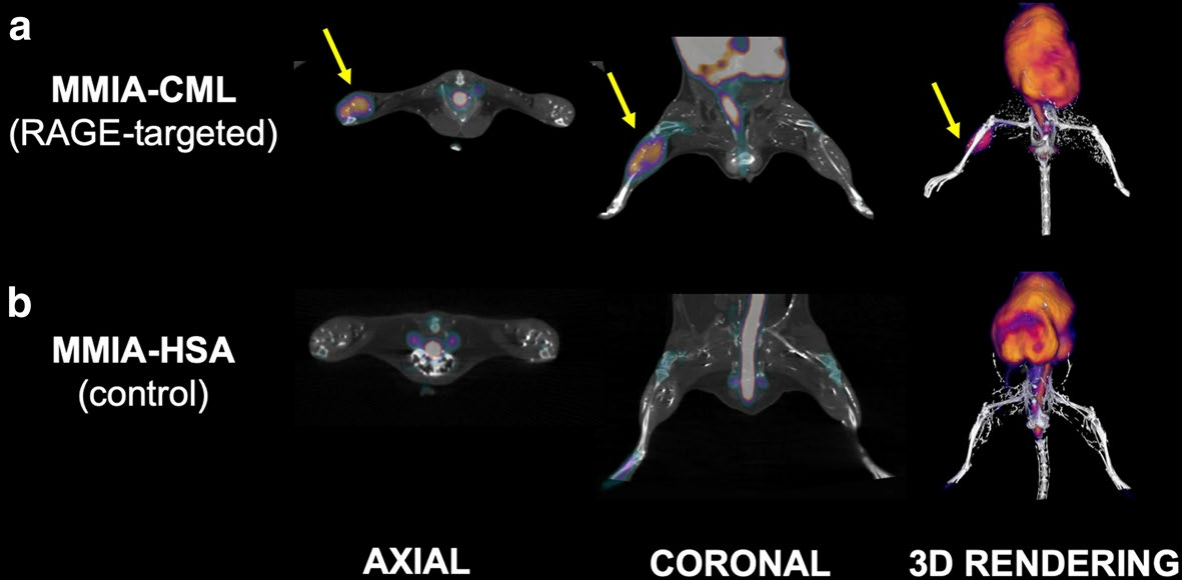
Exemplary PET images of MMIA-CML (**a**) and MMIA-HSA (**b**) nanoparticles radiolabeled with ^64^Cu acquired in a murine model at one week after surgical ligation of the right femoral artery. One hour after radiolabeled probe injection, animals were subjected to the imaging session. Nanoparticle targeted at RAGE notably bonded to the muscle tissue’s ischemic area (yellow arrows) versus the opposite non-ischemic hindlimb; neither ischemic nor non-ischemic hindlimbs exhibited recognizable accumulation of untargeted, control MMIA-HSA

Following in vitro characterization, we examined the RAGE probe’s uptake in a murine model of hindlimb ischemia. One week after femoral artery ligation, mice were injected with MMIA-CML or MMIA-HSA labeled with ^64^Cu radioisotope. Animals subjected to PET imaging exhibited 3.4x greater MMIA-CML uptake in ischemic hindlimbs than non-ischemic hindlimbs. In both ischemic and non-ischemic hindlimbs, the MMIA-HSA probe binding persisted at a low and similar level in both limbs (Fig. 2).

### Ex vivo RAGE expression

**Fig. 3:**
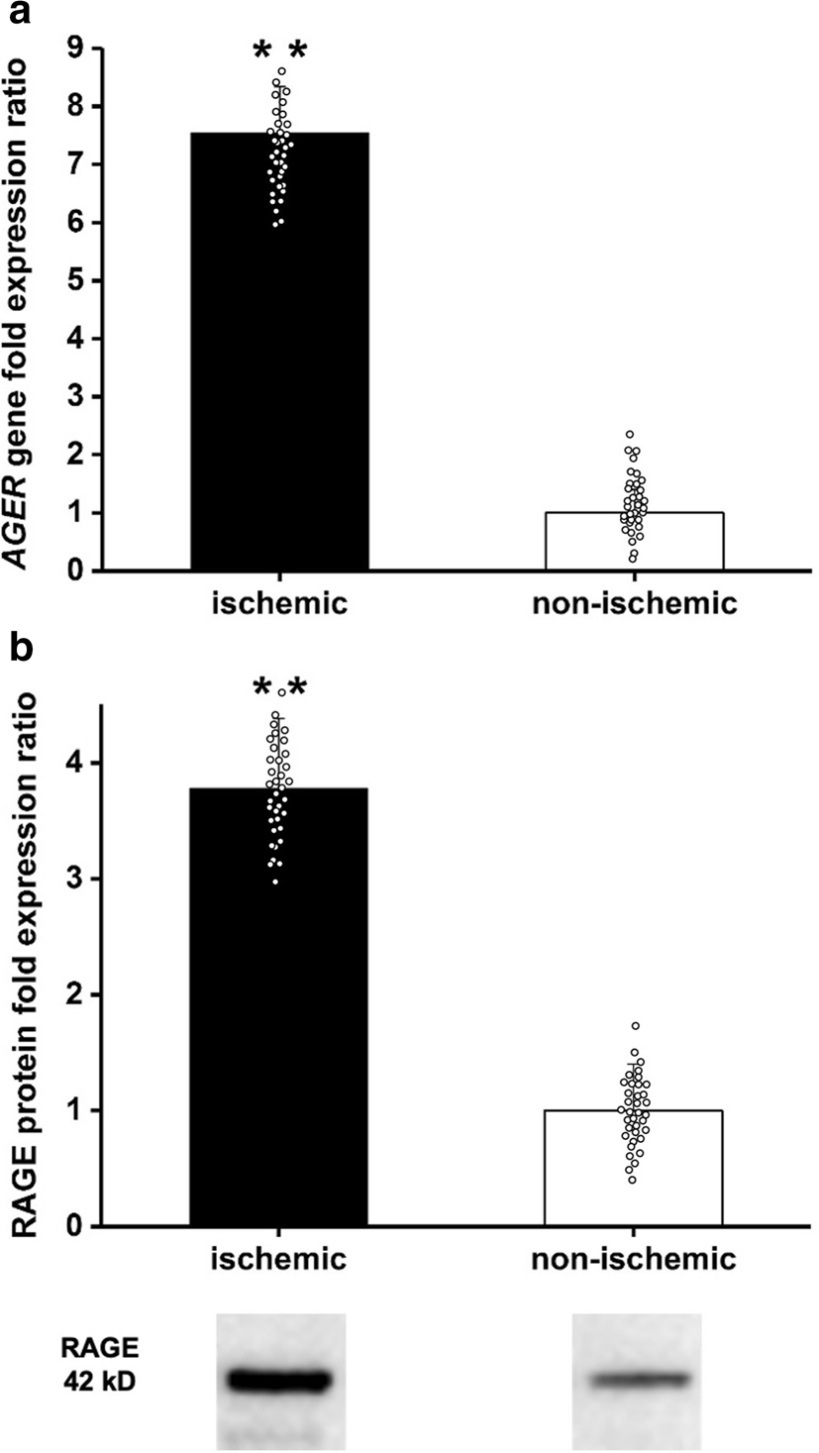
*AGER* (RAGE) gene quantification in murine tissue homogenates from ischemic and non-ischemic hindlimbs at one week after HLi (**a**). Western blot depicting RAGE levels in murine tissue homogenates from ischemic and non-ischemic hindlimbs at one week after HLi (**b**). (**) - *p* < 0.01. Data represent mean and mean squared error

**Fig. 4:**
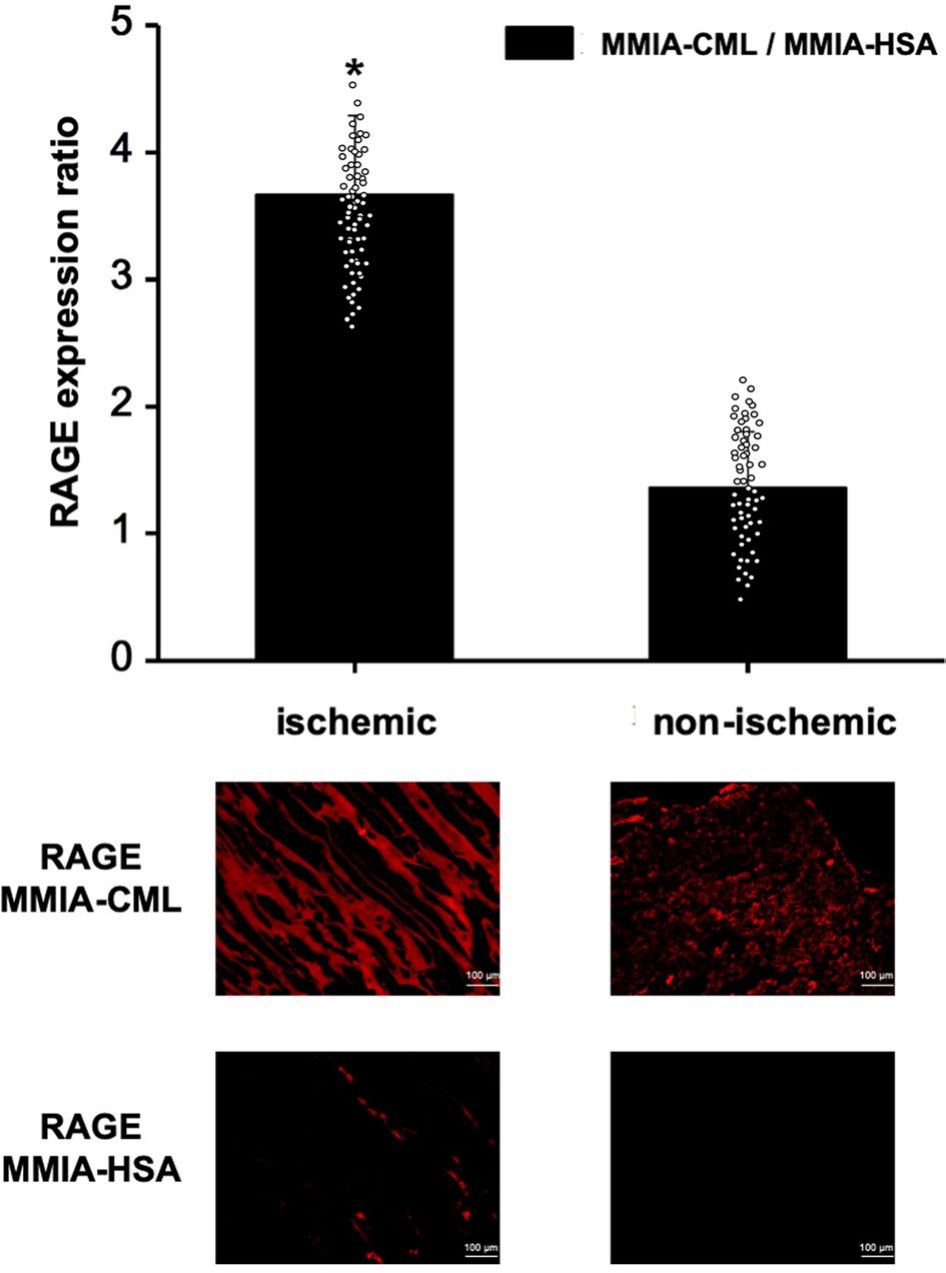
Binding studies using RAGE-targeted (MMIA-CML) and non-targeted (MMIA-HSA) nanoparticles. Murine muscle tissue sections from ischemic and non-ischemic hindlimbs at 1 week after HLi were incubated with 1 µM MMIA-CML or MMIA-HSA. Quantitative measurement proved that ischemic muscle tissue fragments one week after surgical ligation of the femoral artery showed explicit RAGE-targeted probe uptake versus non-ischemic dissected tissue fragments. (*) - *p* < 0.05. Data represent mean and mean squared error

**Fig. 5:**
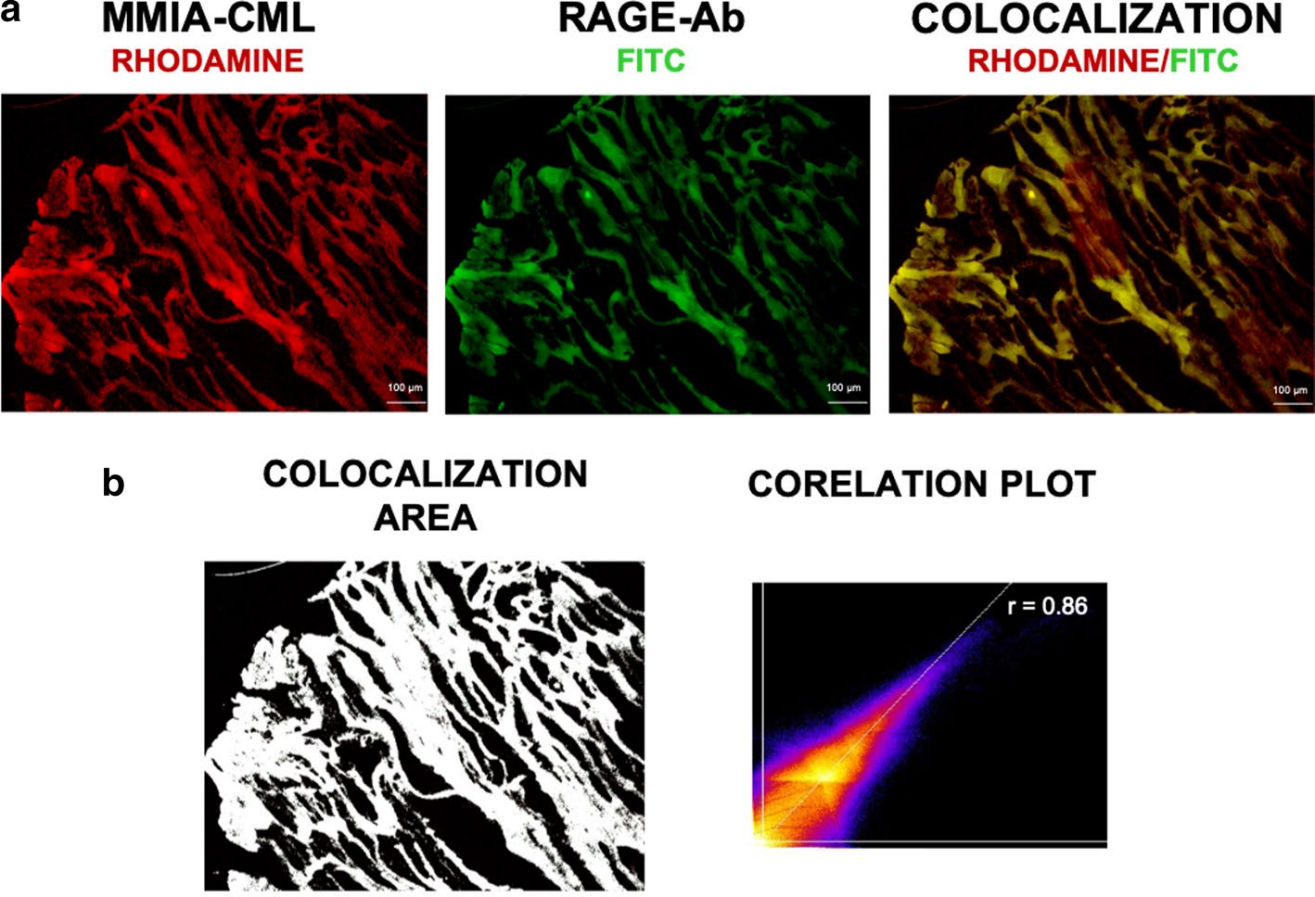
Ex vivo tissue colocalization studies using fluorescence microscopy. Muscle tissue sections from mouse ischemic hindlimbs at one week after HLi were incubated with 1 µM RAGE-targeted nanoparticles and RAGE antibody conjugated with FITC (**a**). Studies demonstrated a significant degree of RAGE-targeted probe and antibody colocalization. Images were analyzed using ImageJ’s Coloc2 plugin and Pearson’s r value was calculated (**b**)

Immediately after PET imaging studies, the murine hindlimb muscle tissues were excised and prepared for gene and protein expression analysis. RAGE gene expression was examined using real-time PCR. RAGE was found to be significantly increased in ischemic hindlimbs one week after ligation as compared to non-ischemic tissue fragments (RAGE gene fold expression ratio: 7.54 ± 0.39) (Fig. 3a). RAGE protein expression was assessed using Western blot technique. Significant RAGE overexpression was observed in ischemic hindlimbs compared to non-ischemic hindlimbs at one week after surgical ligation (RAGE protein fold expression ratio: 3.78 ± 0.40) (Fig. 3b) (Additional file 1). To visualize RAGE expression in excised tissues, we performed fluorescence microscopic imaging using RAGE-targeted and control MMIA. Fluorescence microscopic staining revealed significantly increased uptake of RAGE-targeted nanoparticles in both ischemic and non-ischemic muscle tissues as compared to the control probe but at a higher level in ischemic hindlimbs. Control probe binding remained low and comparable in both ischemic and non-ischemic hindlimbs (Fig. 4). These results were consistent with the observed uptake in PET images. Colocalization analysis between immunofluorescence staining with anti-RAGE antibody and MMIA-CML confirmed this finding. Ischemic tissue fragments exhibited explicit RAGE dyeing following an anti-RAGE antibody and a high degree of colocalization with the MMIA-CML targeted at RAGE (Fig. 5 a, b).

## Discussion

In vivo imaging is a powerful tool for studying complex biological processes both spatially and temporally. Due to the increasing need of the scientific and medical world to simultaneously obtain functional and anatomical data in living organisms, there is a great need to develop molecular probes with multimodal properties that could be utilized with various hybrid imaging techniques. Many multimodal probes combine several sensing capabilities, e.g., PET-MRI. However, imaging with simultaneous combination of PET and optical methods remains a relatively unexplored area [27, 28].

In this paper, the fourth generation PAMAM dendrimer molecule became the backbone structure for our RAGE-targeted molecular probe due to its well-defined, multifunctional surface groups (such as primary amines) enabling conjugation of several different chemical moieties with a slight disturbance of the spherical polymer structure. Furthermore, literature data indicate their successful use as non-invasive imaging agents in the magnetic resonance imaging (MRI), CT and SPECT techniques [27, 29]. Conjugating the NHS-rhodamine fluorochrome and ^64^Cu radioisotope to the PAMAM-based construct ensured the multimodal nature of the synthesized probe. The receptor for advanced glycation end products was selected as the molecular target for the imaging agent due to its importance as an important mediator of the inflammatory process in many pathologies, e.g., atherosclerosis or diabetic complications [30, 31]. Also, a significant relationship has been demonstrated between the incidence of peripheral arterial disease in humans and increased expression of RAGE receptors on endothelial cells [32].

RAGE activation by pro-inflammatory ligands including AGEs (e.g., CML – the best characterized AGE) results in blood vessel damage [33]. The CML-RAGE complex stimulates intracellular signaling pathways responsible for generating reactive oxygen species, reducing the bioavailability of nitric oxide, increasing monocyte and leukocyte chemotaxis and inducing cytokine secretion [30, 31, 34]. To target the RAGE receptor, CML molecule was conjugated with PAMAM dendrimer’s free amine residues.

In our previous work cellular specificity of the synthesized molecular probe was demonstrated using HUVEC cells with induced overexpression of RAGE receptors. In vitro studies were confirmed using in vivo analysis in a murine model of hindlimb ischemia, which is the best-characterized animal model of PAD [35]. Based on the multimodal nature of MMIA, we performed specificity studies to detect both fluorescence intensity and radioactivity. A good correlation of the results obtained using both research techniques was demonstrated.

In the selected experimental model as well as among patients suffering from PAD, there is an initial perfusion impairment, which in consequence leads to hypoxia of the ischemic area. In response to hypoxia, the hypoxia-inducible factor 1-alpha transcription factor locally migrates to the cell nucleus, thereby triggering the signaling pathway leading to vascular endothelial growth factor release and stimulation of angiogenesis through mechanisms affecting the processes of proliferation and apoptosis of endothelial cells and monocyte chemotaxis into ischemic regions [36]. A different study indicated a vital role of microRNAs in modulating nitric oxide bioavailability under hypoxic conditions [34]. It is important that in diabetes, the adaptive response to hypoxia is impaired. The results presented in this article are consistent with literature data on RAGE and angiogenesis-related signaling pathways.

In the case of critical limb ischemia, current diagnostic tests used in clinical practice utilizing routine imaging techniques allow one to detect a blood flow decrease in the vessels and locate the regions of impaired perfusion. Visualization of large vessels such as the aorta is possible e.g., during the autopsy. The assessment of smaller arteries requires the collection of many specimens for histological analysis, and the quantification may be affected by the sampling error. Given the above limitations, an evaluation of the total expression of the receptor or other intracellular structures can be difficult. The implementation of molecular imaging methods with the combination with specific molecular probes in PAD diagnostics could potentially improve the assessment of the extent of lesions within small blood vessels. RAGE and its direct effect on angiogenesis in hypoxic tissue is an important molecular target in PAD. in vivo imaging could provide information on the total vascular expression of RAGE in live individuals, both as a research tool and as a non-invasive marker for the targeted therapy that suppresses RAGE excessive expression – reducing clinical symptoms and improving the prognosis for PAD.

Ritthaler and colleagues demonstrated a significant increase in RAGE expression in the endothelium of small and medium arteries in both diabetic and non-diabetic patients with occlusive peripheral vascular disease [32]. Based on the above data, Tekabe et al. synthesized RAGE-specific molecular probe and proved that it is possible to image and quantify RAGE expression in a diabetic murine model of PAD. Tekabe’s molecular probe was radiolabeled with technetium-99 m (^99 m^Tc) for SPECT imaging and was made specific to RAGE by using monoclonal antibody fragments [37]. The availability of monoclonal antibodies and their excellent ability to recognize and bind with very high affinity and specificity to cellular targets have made them an important element in diagnostics and therapy [38]. Currently, more than 20 monoclonal antibodies have been approved by the US Food and Drug Administration for therapeutic intervention, and several radiolabeled antibody-based probes have been approved for imaging using the SPECT technique [39].

Antibody-based molecular probes have been fundamental in understanding the real-time pharmacokinetics and dynamics of immunoglobulins but have not had a significant impact on clinical practice and patient management. This is due to the high immunogenicity of murine antibodies, which are recognized by the human immune system and initiate a cascade of undesirable side effects. A significant limitation on the use of antibodies in molecular imaging involves their prolonged presence in the blood lasting from several hours to several weeks. This aspect directly affects the results of the analysis. The low detection ratio of the specific signal coming from the molecular probe to the background signal resulted in low-quality scattered images. Limited ability for antibodies to cross biological barriers (e.g., blood-brain barrier, cell membrane) presents an additional challenge that the scientific community needs to address before successfully translating this methodology into clinical practice.

Nanotechnology’s dynamic evolution has enabled the development of many materials used in molecular imaging probes’ synthesis and helped to overcome many experimental challenges [40–42]. Among other available nanomaterials, dendrimers characterized by a branched, well-defined composition and three-dimensional structure have aroused researchers’ broad interest. Dendrimers have been successfully used in many biomedical applications thanks to their unique parameters, including variable size and ability to modify surface chemical groups [43–45]. The potential to functionalize the dendrimers with bioactive ligands strengthens their cellular specificity; one prime example is the RAGE-targeted molecular imaging agent developed by our group [24].

In contrast, the use of dendrimers and other nanoparticles as platforms for the construction of molecular imaging probes has a number of disadvantages. Nanoparticles require the presence of a stable coating capable of chemical functionalization. Many nanoparticles exhibit long tissue retention, and knowledge about their cellular toxicity is insufficient (e.g., quantum dots made of cadmium ions or other toxic metals). An additional challenge is the lack of full control over the stoichiometric reaction of coupling selected chemical moieties on the dendrimer surface and quantitative assessment of the degree of conjugation. Nevertheless, most of the designed molecular probes with multimodal properties, in particular those used for PET and optical imaging, are based on nanoparticles.

In principle, there is a possibility to combine PET and optical imaging methods using separate molecular markers for each of these techniques instead of a hybrid imaging marker. However, this is not an optimal strategy considering the different biodistribution and pharmacokinetic properties of the two probes. Therefore, in the present study to obtain reliable and comparable results between the radioisotope and optical imaging method, a hybrid imaging agent specific for RAGE labeled with radionuclide and fluorescent dye was developed.

Although optical imaging, due to the low light tissue penetration, has limited use for in vivo imaging of entire organisms, it could be a valuable tool in image-guided surgery (IGS), enabling intraoperative localization of anatomical structures with high spatial resolution and specificity [46, 47]. In oncological surgery, the combination of PET imaging using deep-penetrating gamma radiation to localize the tumor and subsequent intraoperative optical imaging to identify tumor margins and assess adjacent lymph nodes could significantly improve the patient’s clinical situation [48, 49]. Importantly, imaging using optical techniques is not fully quantifiable due to the absorption and scattering of photons by tissue, so it cannot be fully correlated with data obtained using radiological techniques [7, 50, 51]. Therefore, when locating a tumor lesion or other anatomical structure during IGS, quantitative analysis is not decisive. However, taking into account the above limitations and challenges, the future of molecular imaging is very optimistic. Further development of molecular imaging can lead to significant breakthroughs in understanding biological processes and mechanisms occurring in vivo.

### The bottleneck of our study

Molecular imaging has revolutionized how we analyze fundamental biological processes and diagnose and monitor certain diseases. However, to realize the full potential of molecular imaging, several difficulties and many challenges must be overcome. The Achilles’ heel of most molecular imaging techniques is undoubtedly the need to administer an exogenous imaging agent to evaluate selected biochemical processes. Molecular probe development is a relatively long, tedious, and costly investment that rarely produces clinically useful results and brings financial benefits. Currently, in molecular imaging, there is a minimal number of scientists and clinicians with multidisciplinary education. Most have a solid foundation in one or two areas and are specialized in handling specific devices using a specific method. Therefore, molecular imaging requires an interdisciplinary approach by scientists to a research problem in order to understand it using imaging techniques. A molecular probe’s usefulness in clinical applications should be demonstrated to translate experiments with prospective molecular probes into medical practice. Close collaboration between academic groups involved in development of molecular imaging tracers is needed to accelerate translational research and conduct multi-site international clinical trials. It is also essential to develop a model of cooperation between the scientific community and industry, which will make it easier to conduct pilot clinical trials on promising molecular probes.

## Conclusions

Our study illustrates the utility of the synthesized molecular probe targeted to RAGE, as an imaging agent in both in vivo PET imaging and in vitro and ex vivo fluorescence microscopy. In HUVEC with induced RAGE overexpression incubated with RAGE-targeted MMIA-CML, we found a strong correlation between fluorescent signal detection and radioactivity measurement. Moreover, in post-HLi animals, we observed overexpression of RAGE at the gene and protein level and demonstrated strong colocalization using anti-RAGE antibody and RAGE-targeted MMIA-CML. The present results indicate that successful combination of in vitro diagnostics and molecular imaging would help better identify the population at risk of pathology. Moreover, in our recent paper we presented a novel preclinical multimodal imaging approach focused on the RAGE receptor to diagnose and track prostate cancer progression [52]. Our research revealed that RAGE’s imaging strategy is achievable and can reshape prostate cancer treatment [52]. Effective screening using non-invasive based imaging methods and routine diagnostic techniques would improve early detection of the disease long before the clinical manifestation and first symptoms occur, possibly leading to the design and application of an individualized therapeutic strategy. The fusion of both in vitro and in vivo diagnostics will reduce the limitations of each approach and simultaneously positively affect the development of personalized medicine.

## Supplementary Information

**Additional file 1. **Additional figures.

## Data Availability

The datasets used and/or analysed during the current study are available from the corresponding author on reasonable request.

## Abbreviations

*ACTB*: Beta-actin protein coding gene
AGEs: Advanced glycation end products
*AGER*: RAGE protein coding gene
CML: Nε-carboxymethyl-lysine
DNA: Deoxyribonucleic acid
FBS: Fetal bovine serum
*GAPDH*: Glyceraldehyde 3-phosphate dehydrogenase protein coding gene
HLi: Hindlimb ischemia
HSA: Human serum albumin
HUVEC: Human umbilical vein endothelial cells
IGS: Image-guided surgery (IGS)
microPET-CT: Micro positron emission tomography combined with computed tomography
MMIA: Multimodal imaging agent
MMIA-CML: Multimodal imaging agent conjugated with CML
MMIA-HSA: Multimodal imaging agent conjugated with HSA
MRI: Magnetic resonance imaging
PAMAM dendrimer: Polyamidoamine dendrimer
PAD: Peripheral artery disease
PCR: Polymerase chain reaction
PET: Positron emission tomography
RAGE: Receptor for advanced glycation end products
RNA: Ribonucleic acid
SPECT: Single-photon emission computed tomography
TBST: Tris buffered saline with Tween
VEGF: Vascular endothelial growth factor
