# Supplementary material for: Molecularly targeted nanoparticles: an emerging tool for evaluation of expression of the receptor for advanced glycation end products in a murine model of peripheral artery disease
Source: Cell Mol Biol Lett. 2021 Mar 16;26:10. doi: 10.1186/s11658-021-00253-0 (PMC7968326; doi:10.1186/s11658-021-00253-0)
Supplement: Supplementary file 1 — Additional file 1. Additional figures. [file 11658_2021_253_MOESM1_ESM.pdf]

Supplementary material

In the postnatal period, the expression levels of RAGE remains low in almost all normal tissues [1-3], and the possible temporal increase in a RAGE expression is relevant to the response to the ongoing pathological processes [4]. The exceptions are the lungs and skin, where expression remains high throughout life.

Supplementary Figure 1 represents western blots contributed to the quantitative RAGE expression analysis in response to HLi in Figure 3B.

Supplementary Figure 2 shows analysis of anti-RAGE antibody specificity in murine lungs (A) - positive control, and pre-adsorption with anti-RAGE antibody binding peptide (B,C) - negative control.

Figure 1. Supplementary Figures 1A, B, C, D, E and F represent western blots contributed to the quantitative analysis shown in Figure 3B**.** Western blot (with using primary Abcam antibody, catalog no ab30381) depicting RAGE levels in murine tissue homogenates from ischemic and non-ischemic hindlimbs at one week after HLi. Experiments were performed in triplicates.

Fig. 1A

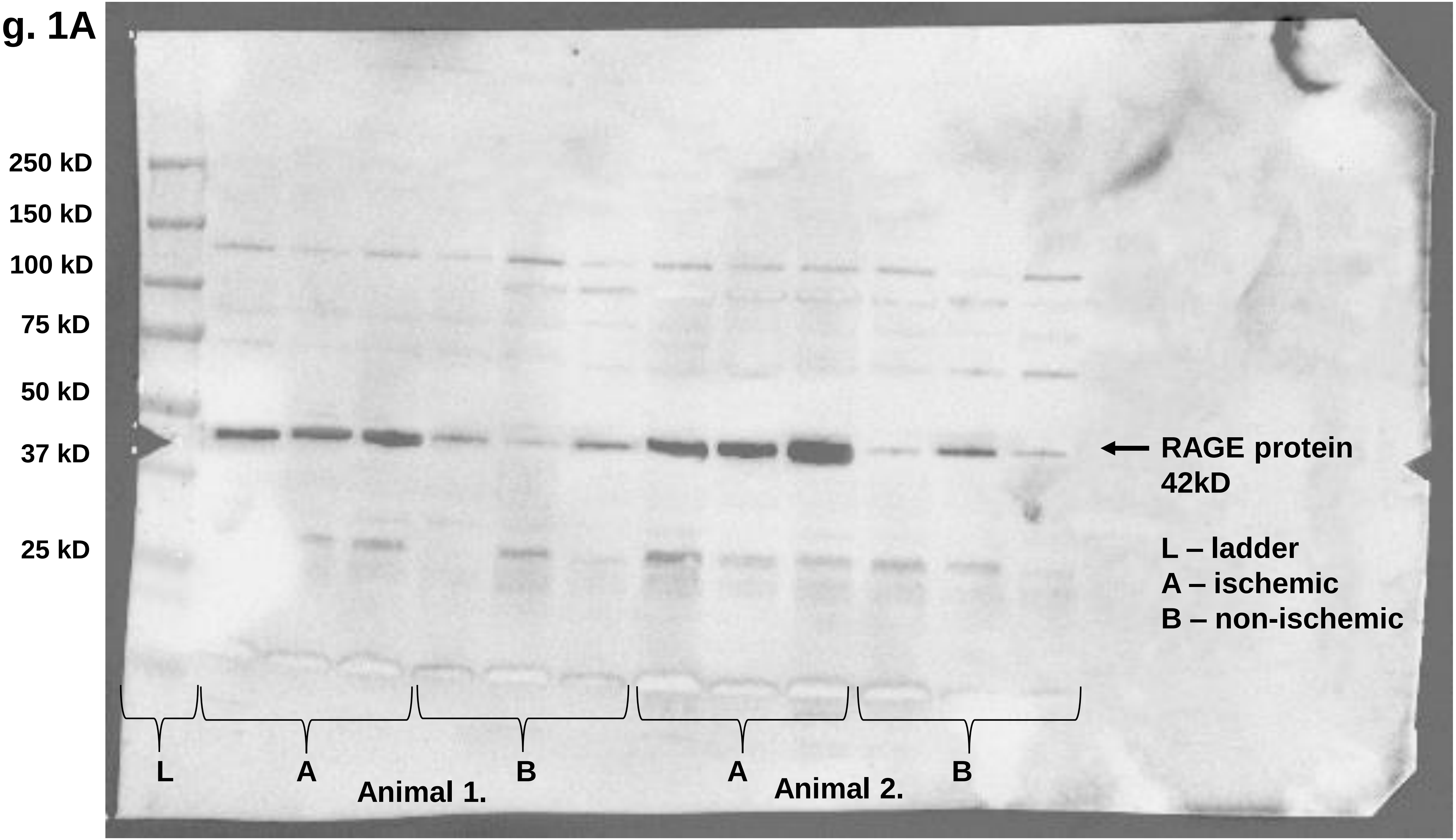
250 kD
150 kD
100 kD
75 kD
50 kD
37 kD
25 kD
RAGE protein
42kD
L – ladder
A – ischemic
B – non-ischemic
L
A
B
A
B
Animal 1.
Animal 2.

Fig. 1B

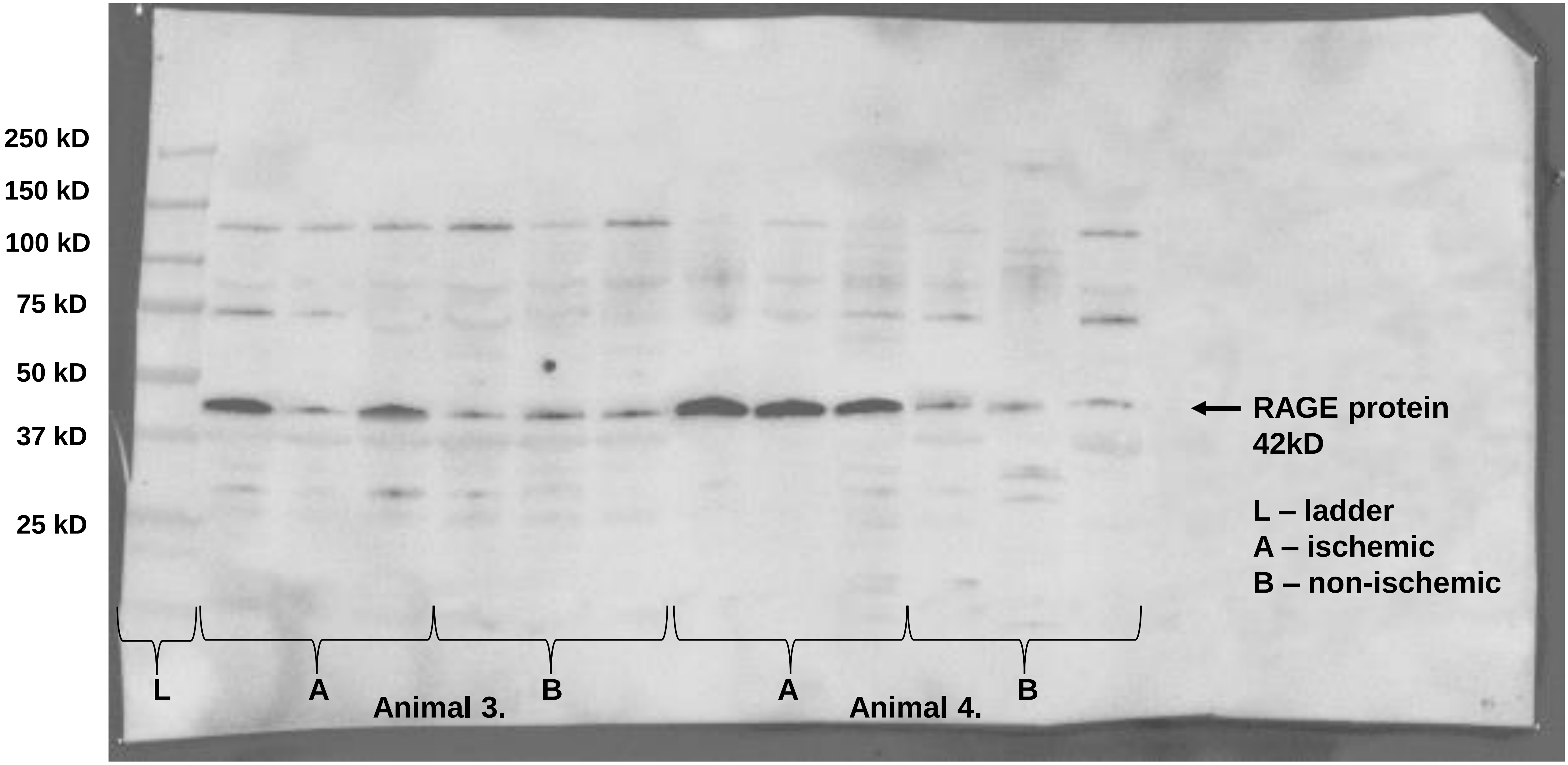

250 kD
150 kD
100 kD
75 kD
50 kD
37 kD
25 kD
RAGE protein
42kD
L – ladder
A – ischemic
B – non-ischemic
L
A
B
Animal 3.
A
B
Animal 4.

Fig. 1C

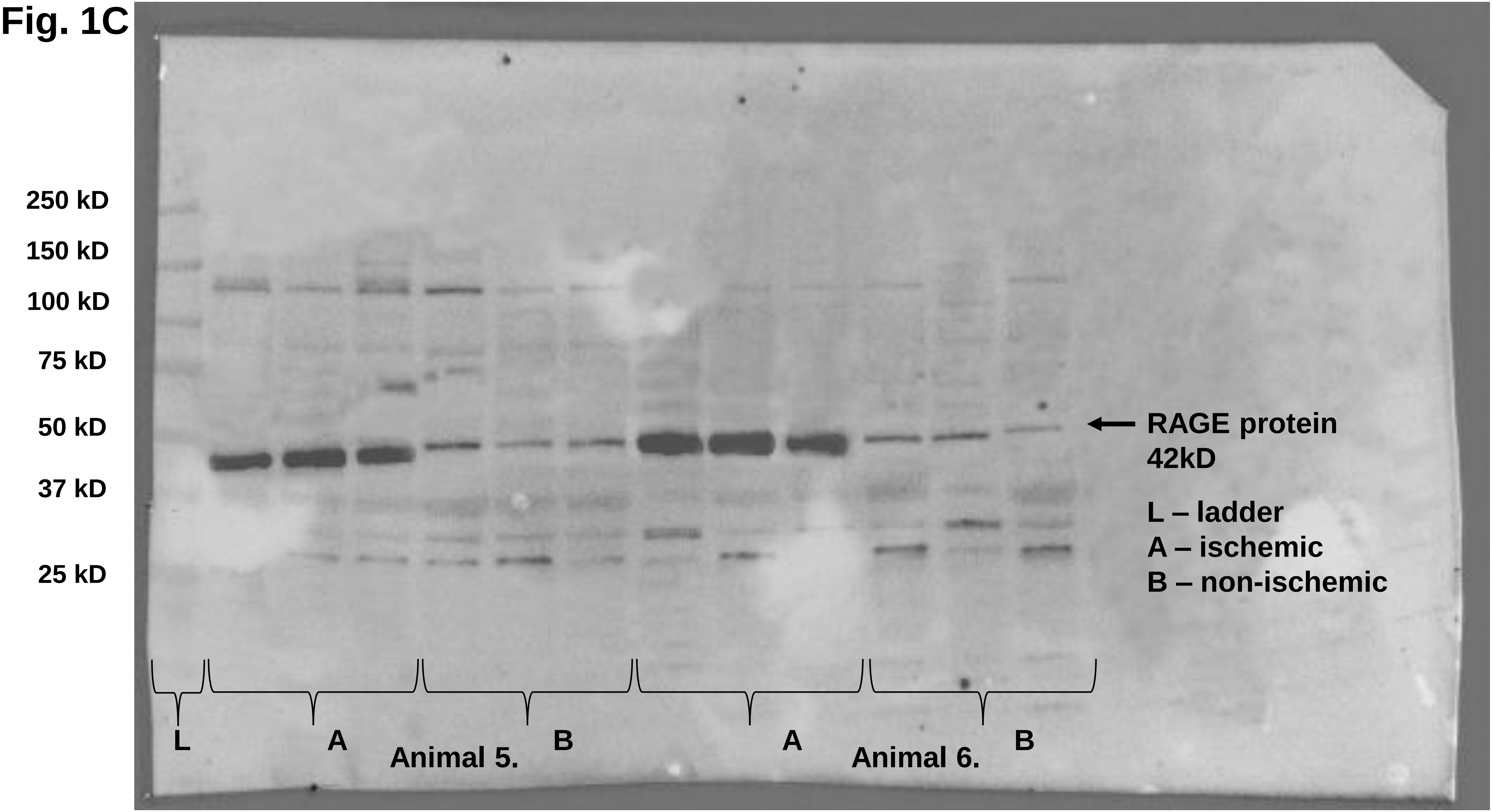

250 kD
150 kD
100 kD
75 kD
50 kD
37 kD
25 kD
RAGE protein
42kD
L – ladder
A – ischemic
B – non-ischemic
L
A
Animal 5.
B
A
Animal 6.
B

Fig. 1D

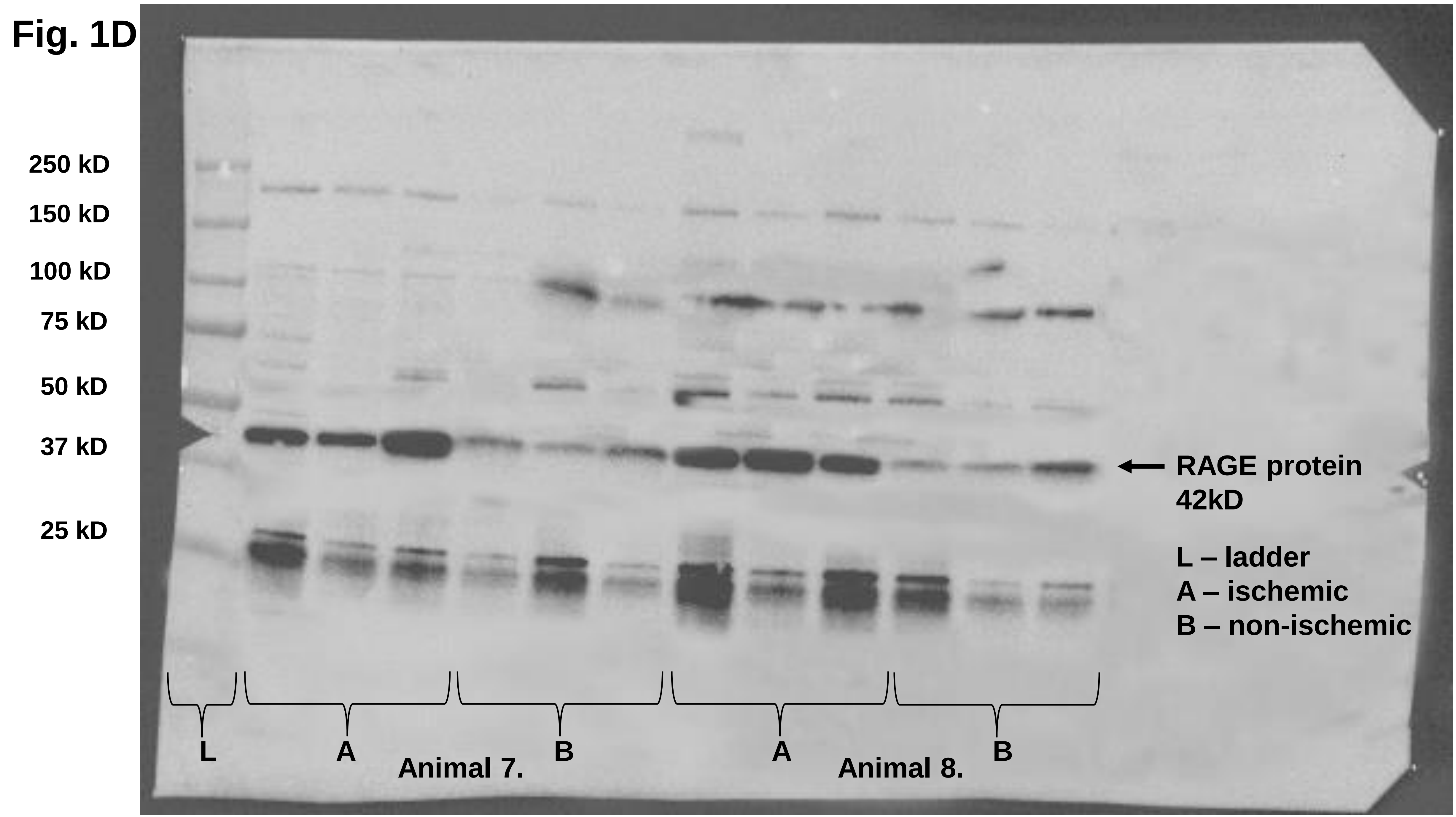
250 kD
150 kD
100 kD
75 kD
50 kD
37 kD
25 kD
RAGE protein
42kD
L – ladder
A – ischemic
B – non-ischemic
L
A
Animal 7.
B
A
Animal 8.
B

Fig. 1E

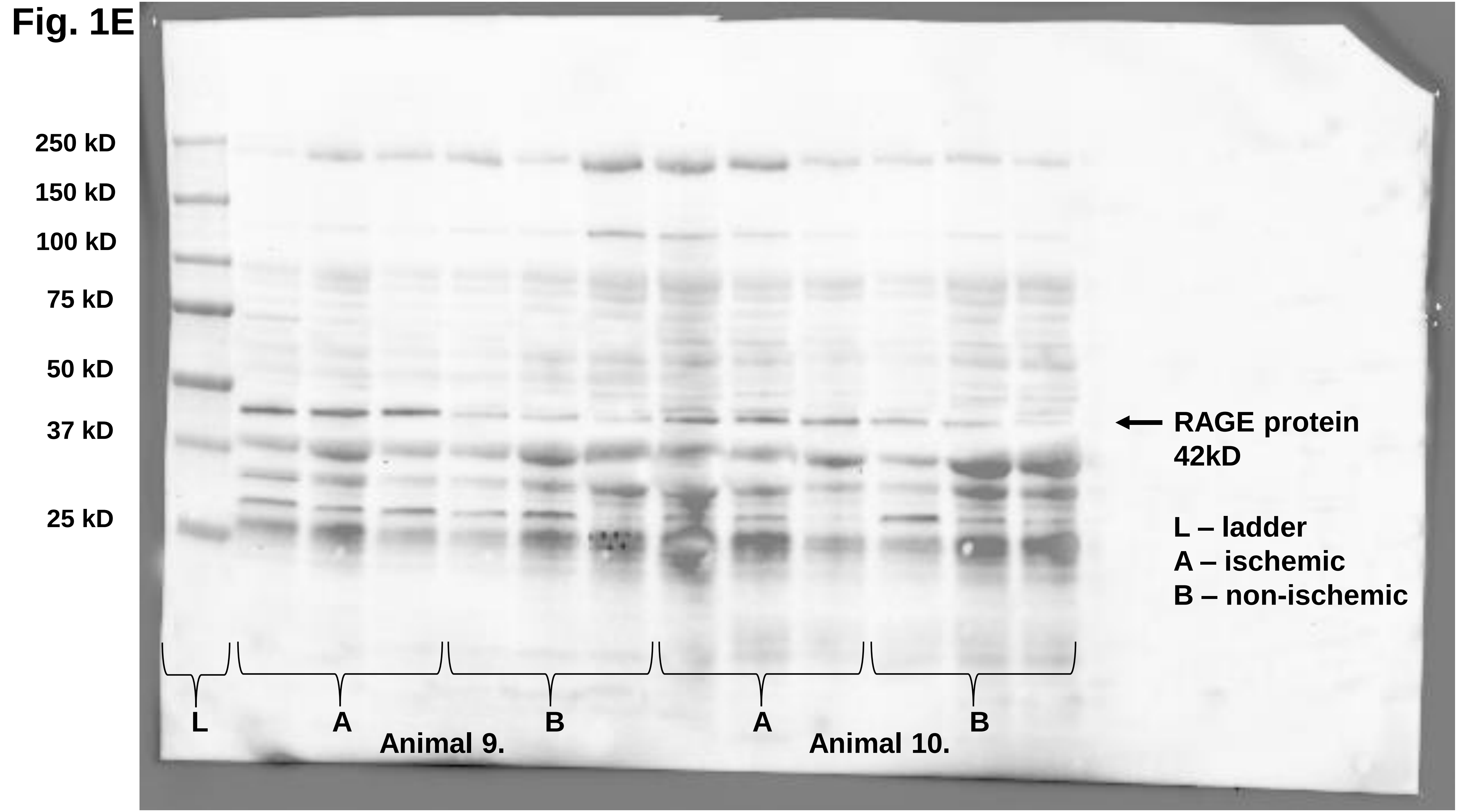
250 kD
150 kD
100 kD
75 kD
50 kD
37 kD
25 kD
RAGE protein
42kD
L – ladder
A – ischemic
B – non-ischemic
L
A
B
A
B
Animal 9.
Animal 10.

Fig. 1F

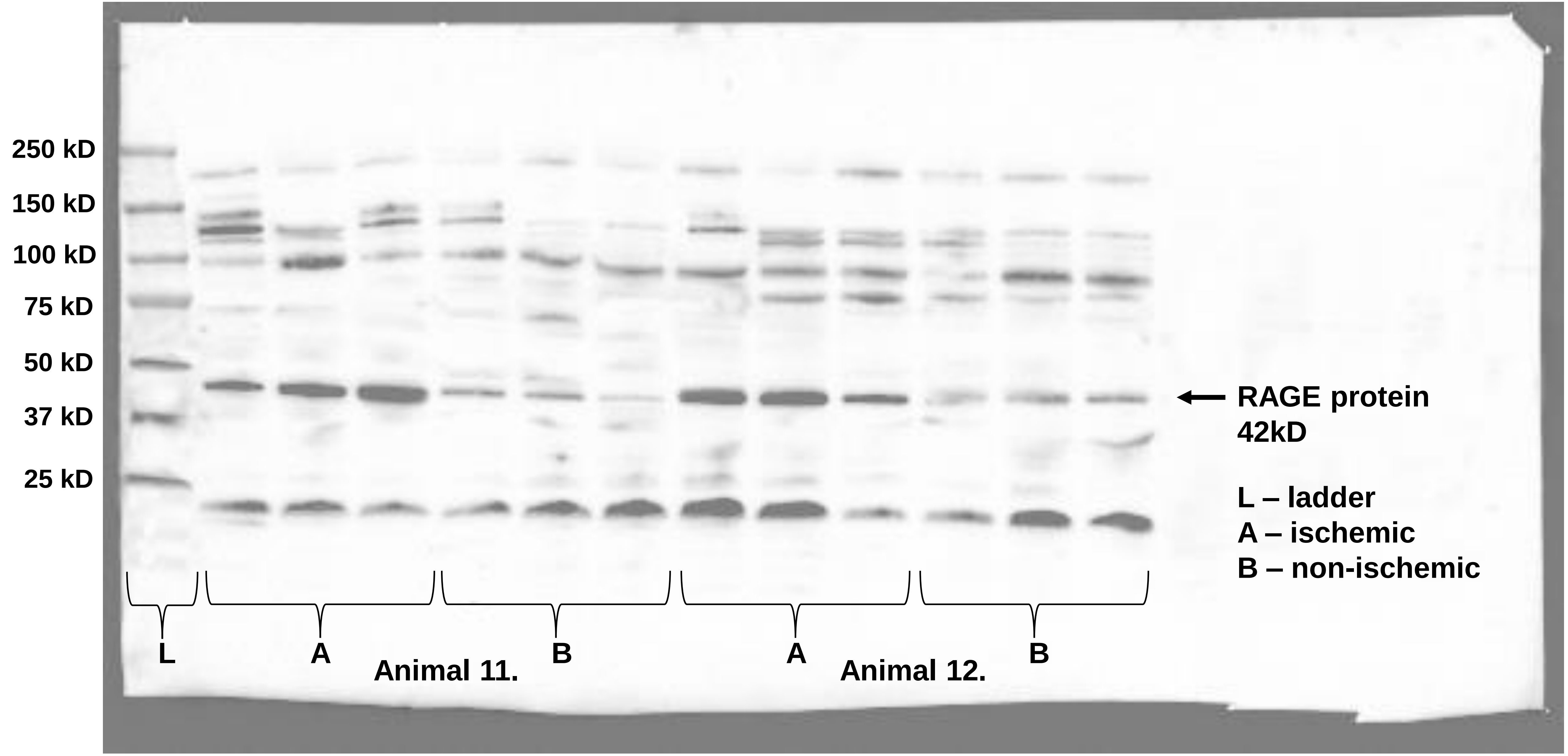
250 kD
150 kD
100 kD
75 kD
50 kD
37 kD
25 kD
RAGE protein
42kD
L – ladder
A – ischemic
B – non-ischemic
L
A
Animal 11.
B
A
Animal 12.
B

Figure 2. Analysis of anti-RAGE antibody (Abcam antibody, catalog no ab30381) specificity using RAGE reference tissue (A) - positive control, and anti-RAGE antibody binding peptide (B,C) - negative control.

Supplementary Figure 2A represents RAGE protein expression in murine lung tissue in the studied animals (1-12). Murine lung tissue extracts were used to confirm the anti-RAGE antibody's specificity. Lung tissue fragments were collected from each animal, and homogenates were prepared for western blot analysis.

Supplementary Figures 2B (studied animals 1-8) and C (studied animals 9-12) show no RAGE protein expression in murine tissue homogenates after pre-adsorption with anti-RAGE antibody binding peptide (Abcam, catalog no ab32414) from ischemic and non-ischemic hindlimbs at one week post HLi. The anti-RAGE antibody was pre-incubated with an excess of the anti-RAGE antibody binding peptide. Pre-adsorption decreases the intensity of RAGE protein staining corresponding to the RAGE protein expression band.

Fig. 2A

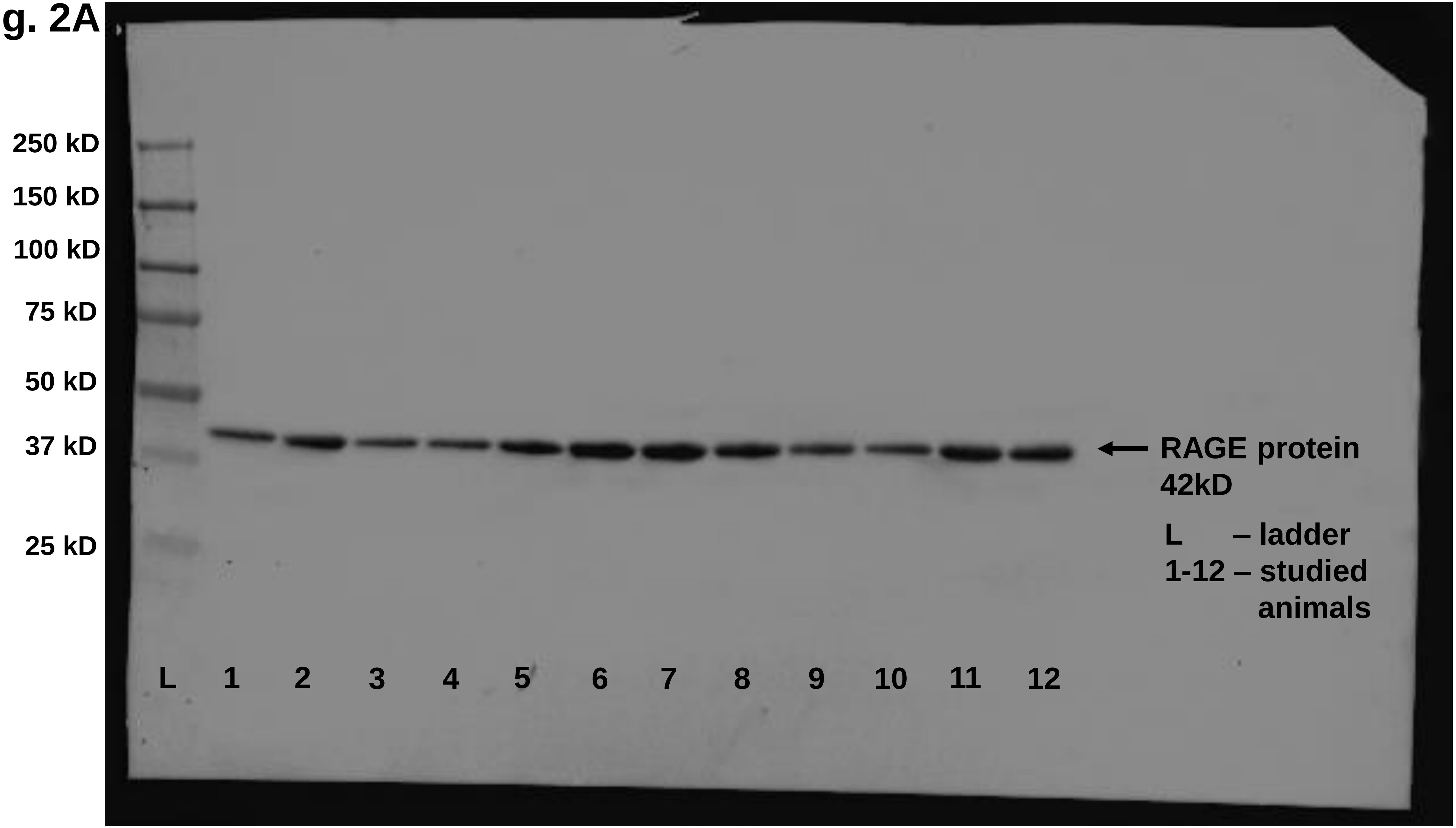
250 kD
150 kD
100 kD
75 kD
50 kD
37 kD
25 kD
RAGE protein
42kD
L – ladder
1-12 – studied animals
L 1 2 3 4 5 6 7 8 9 10 11 12

Fig. 2B

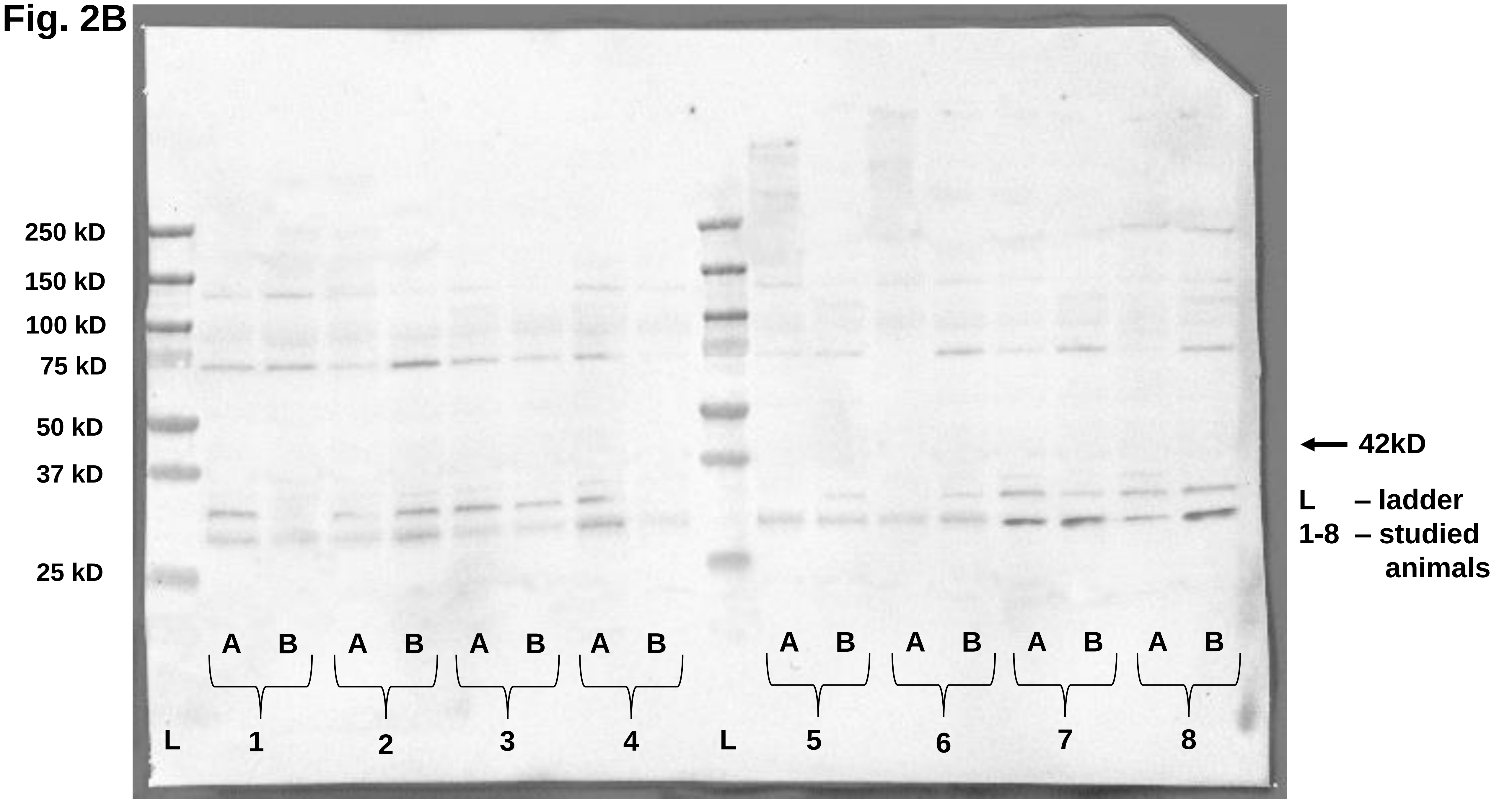
250 kD
150 kD
100 kD
75 kD
50 kD
37 kD
25 kD
A B A B A B A B
L 1 2 3 4
L
A B A B A B A B
5 6 7 8
42kD
L – ladder
1-8 – studied animals

Fig. 2C

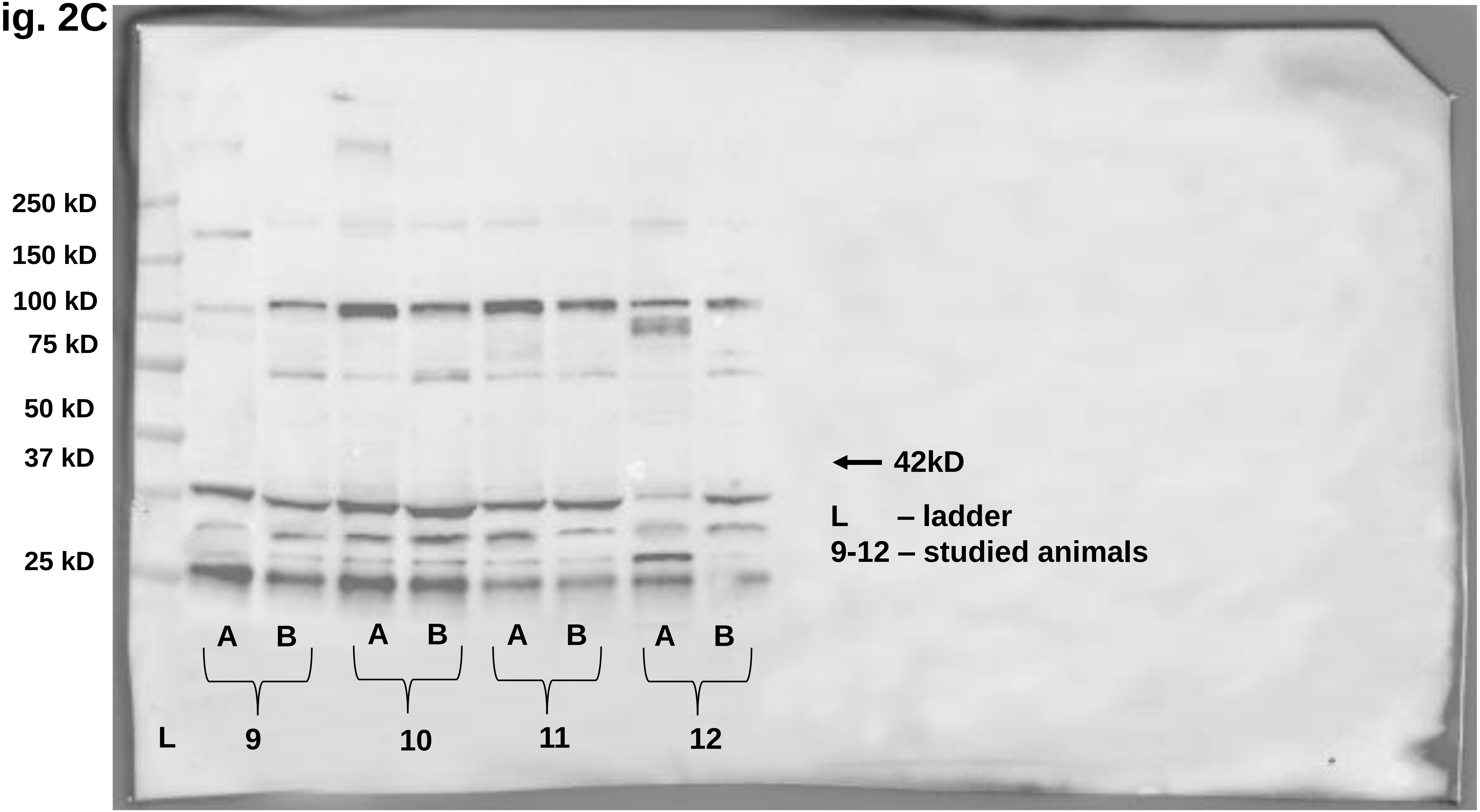
250 kD
150 kD
100 kD
75 kD
50 kD
37 kD
25 kD
42kD
L – ladder
9-12 – studied animals
A B A B A B A B
L 9 10 11 12

References

1. Grillo MA, Colombatto S. Advanced glycation end-products (AGEs): Involvement in aging and in neurodegenerative diseases. Amino Acids. 2008;35(1):29–36.
2. Semba RD, Nicklett EJ, Ferrucci L. Does accumulation of advanced glycation end products contribute to the aging phenotype? Journals Gerontol - Ser A Biol Sci Med Sci. 2010;65 A(9):963–75.
3. Brett J, Schmidt AM, Shi Du Yan, Yu Shan Zou, Weidman E, Pinsky D, et al. Survey of the distribution of a newly characterized receptor for advanced glycation end products in tissues. Am J Pathol. 1993;143(6):1699–712.
4. Ramasamy R, Yan SF, Schmidt AM. RAGE: therapeutic target and biomarker of the inflammatory response-the evidence mounts. J Leukoc Biol. 2009;86(3):505–12.
